# High performance of graphene oxide-doped silicon oxide-based resistance random access memory

**DOI:** 10.1186/1556-276X-8-497

**Published:** 2013-11-21

**Authors:** Rui Zhang, Kuan-Chang Chang, Ting-Chang Chang, Tsung-Ming Tsai, Kai-Huang Chen, Jen-Chung Lou, Jung-Hui Chen, Tai-Fa Young, Chih-Cheng Shih, Ya-Liang Yang, Yin-Chih Pan, Tian-Jian Chu, Syuan-Yong Huang, Chih-Hung Pan, Yu-Ting Su, Yong-En Syu, Simon M Sze

**Affiliations:** 1 School of Software and Microelectronics, Peking University, Beijing 100871, People's Republic of China; 2 Department of Materials and Optoelectronic Science, National Sun Yat-Sen University, Kaohsiung 804, Taiwan; 3 Department of Physics, National Sun Yat-Sen University, Kaohsiung 804, Taiwan; 4 Advanced Optoelectronics Technology Center, National Cheng Kung University, Tainan 700, Taiwan; 5 Department of Electronic Engineering and Computer Science, Tung-Fang Design Institute, Kaohsiung, Taiwan; 6 Department of Chemistry, National Kaohsiung Normal University, Kaohsiung, Taiwan; 7 Department of Mechanical and Electro-Mechanical Engineering, National Sun Yat-Sen University, Kaohsiung, Taiwan; 8 Department of Electronics Engineering, National Chiao Tung University, Hsinchu, Taiwan

**Keywords:** High performance, Graphene oxide, RRAM, Hopping conduction

## Abstract

In this letter, a double active layer (Zr:SiO_*x*_/C:SiO_*x*_) resistive switching memory device with outstanding performance is presented. Through current fitting, hopping conduction mechanism is found in both high-resistance state (HRS) and low-resistance state (LRS) of double active layer RRAM devices. By analyzing Raman and FTIR spectra, we observed that graphene oxide exists in C:SiO_*x*_ layer. Compared with single Zr:SiO_*x*_ layer structure, Zr:SiO_*x*_/C:SiO_*x*_ structure has superior performance, including low operating current, improved uniformity in both set and reset processes, and satisfactory endurance characteristics, all of which are attributed to the double-layer structure and the existence of graphene oxide flakes formed by the sputter process.

## Background

Recently, the applications of mobile electronic products, such as combined display designs [1-9], memories [10-12], and logic ICs, have popularized considerably. With the growing demand of powerful mobile electronic products, non-volatile memory (NVM) has been widely applied due to its low power consumption requirements. To surmount the technical and physical limitation issues of conventional charge storage-based memories [13-17], the resistance random access memory (RRAM) is a kind of promising NVM due to its superior characteristics such as low cost, simple structure, high-speed operation, non-destructive readout, and the compatibility in the semiconductor industry [18-39].

Graphene and graphene oxide-based materials attract vast attention and have been applied into various fields [40]. Graphene oxide (GO) is a material of great interest for its special quality, and its electrical properties can be modified by altering the attached chemical groups. It exhibits resistance switching behaviors by adding and removing oxygen-containing groups, which are quite different from common filament dominant resistance switching [41-44].

In our research, double resistive switching layer RRAM with a sandwiched structure of Pt/Zr:SiO_*x*_/C:SiO_*x*_/TiN was fabricated to investigate the switching merits by inserting C:SiO_*x*_ layer. Graphene oxide was observed in the inserted layer from the analysis of Raman and Fourier transform infrared (FTIR) spectra. Meanwhile, single resistive switching layer devices (Pt/Zr:SiO_*x*_/TiN) were also fabricated so as to make a comparison. Through current fitting, hopping conduction mechanism was found in both high-resistance state (HRS) and low-resistance state (LRS) of Zr:SiO_*x*_/C:SiO_*x*_ RRAM devices. The resistance switching properties of graphene oxide was different from unstable metal filament formation and rupture [45,46]. The performance of RRAM devices has always been one of the targets which influence its mass production and wide application in the semiconductor industry. This is also the reason why the performance of Zr:SiO_*x*_/GO:SiO_*x*_ stacking structure is focused and analyzed in detail in this paper owing to its superior properties from various aspects.

## Methods

The experimental specimens were prepared as follows: for the single active layer specimen, the Zr:SiO_*x*_ thin film (about 20 nm) was deposited on the TiN/Ti/SiO_2_/Si substrate by co-sputtering with the pure SiO_2_ and Zr targets. The active layer was deposited onto patterned TiN bottom electrode, and the sputtering power was fixed at RF power 200 and 20 W for SiO_2_ and Zr targets, respectively. The co-sputtering was executed in argon ambient (Ar = 30 sccm) with a working pressure of 6 mTorr at room temperature. However, for the double resistive switching layer specimen, first a C:SiO_*x*_ film (about 6 nm) was deposited by co-sputtering with the SiO_2_ and C targets. The sputtering power was fixed at RF power 200 and 5 W for SiO_2_ and C targets, respectively. The co-sputtering was also executed in argon ambient (Ar = 30 sccm) with a working pressure of 6 mTorr at room temperature. Then, the layer of Zr:SiO_*x*_ (about 14 nm) was deposited with the same RF power, argon ambient, and working pressure as antecedent single Zr:SiO_*x*_ layer specimen.

Ultimately, the Pt top electrode of 200-nm thickness was deposited on both specimens by direct current (DC) magnetron sputtering. The entire electrical measurements of devices with the Pt electrode of 250-μm diameter were performed using Agilent B1500 semiconductor parameter analyzer (Santa Clara, CA, USA). Besides, X-ray photoelectron spectroscopy (XPS), FTIR, and Raman spectroscopy were used to analyze the mole fraction, chemical composition, and bonding of these insulator materials, respectively.

## Results and discussion

**Figure 1:**
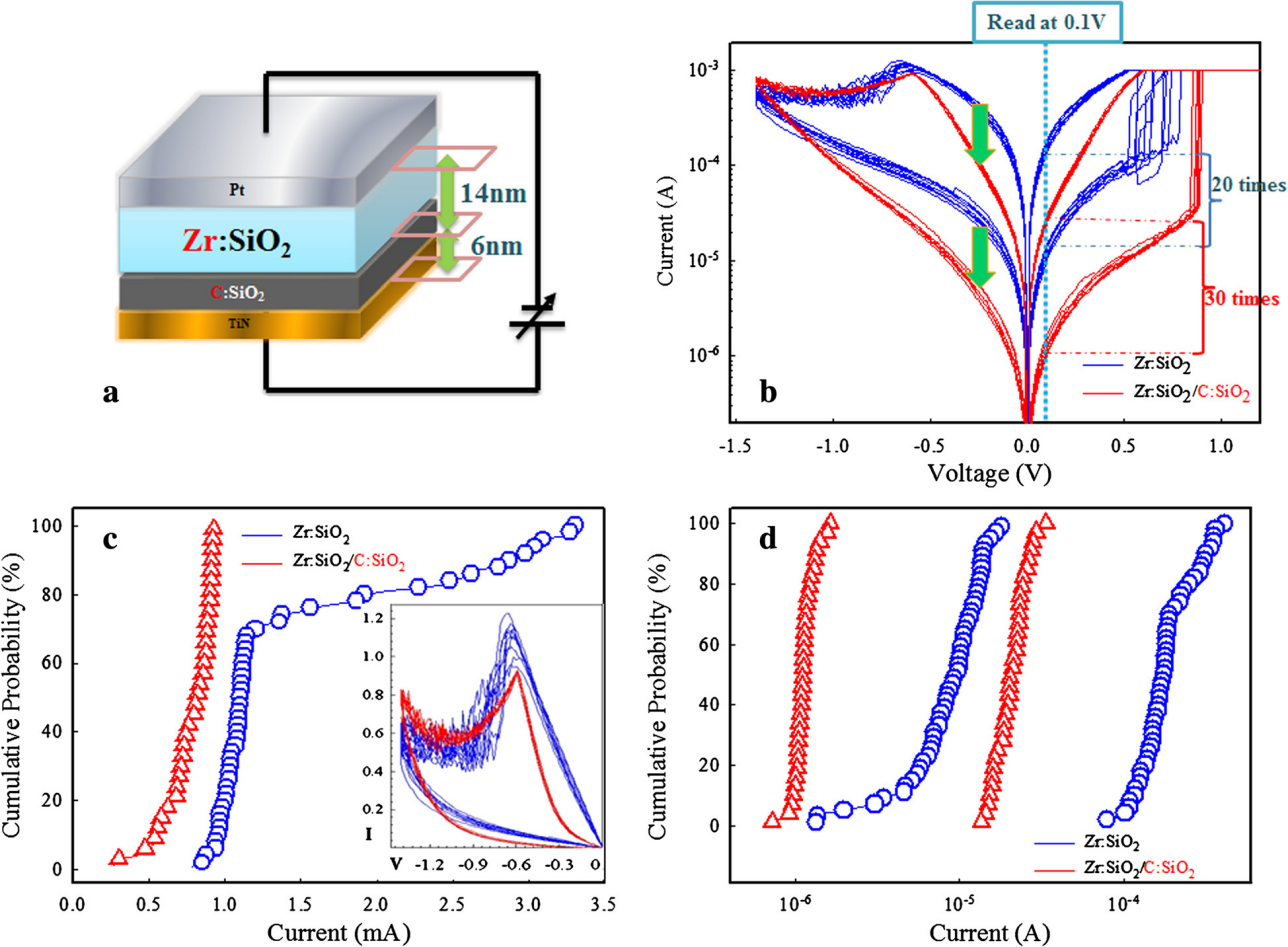
**RRAM device, resistive switching characteristic, reset voltage distributions, and distributions of HRS and LRS. (a)** The RRAM device schematic structure. **(b)** Resistive switching characteristic comparison of single and double switching layer RRAM. **(c)** Comparison of reset voltage distributions. The lower inset shows the corresponding *I*-*V* curve of reset process in linear scale. **(d)** Distributions of HRS and LRS of Zr:SiO_2_ and Zr:SiO_2_/C:SiO_2_ RRAM devices.

A forming process using DC voltage sweeping with a compliance current of 10 μA is required to activate all of the RRAM devices. Afterwards, the DC voltage sweeping cycling test is performed to evaluate both types of devices. Figure 1b shows that Zr:SiO_*x*_/C:SiO_*x*_ RRAM devices exhibit smaller working current on both LRS and HRS. It is noted that the single Zr:SiO_*x*_ layer device shows less attractive characteristics during DC sweeping cycles, including smaller ratio between HRS and LRS, unstable set voltage, and lower degree of uniformity in reset process. If we define the read voltage 0.1 V, the on/off ratios of single- and double-layer devices is 20 and 30, respectively. Meanwhile, from Figure 1c,d, we can see that both the reset voltage and stability between HRS and LRS of Pt/Zr:SiO_*x*_/TiN RRAM show wider distributions compared with Pt/Zr:SiO_*x*_/C:SiO_*x*_/TiN structure devices.

**Figure 2:**
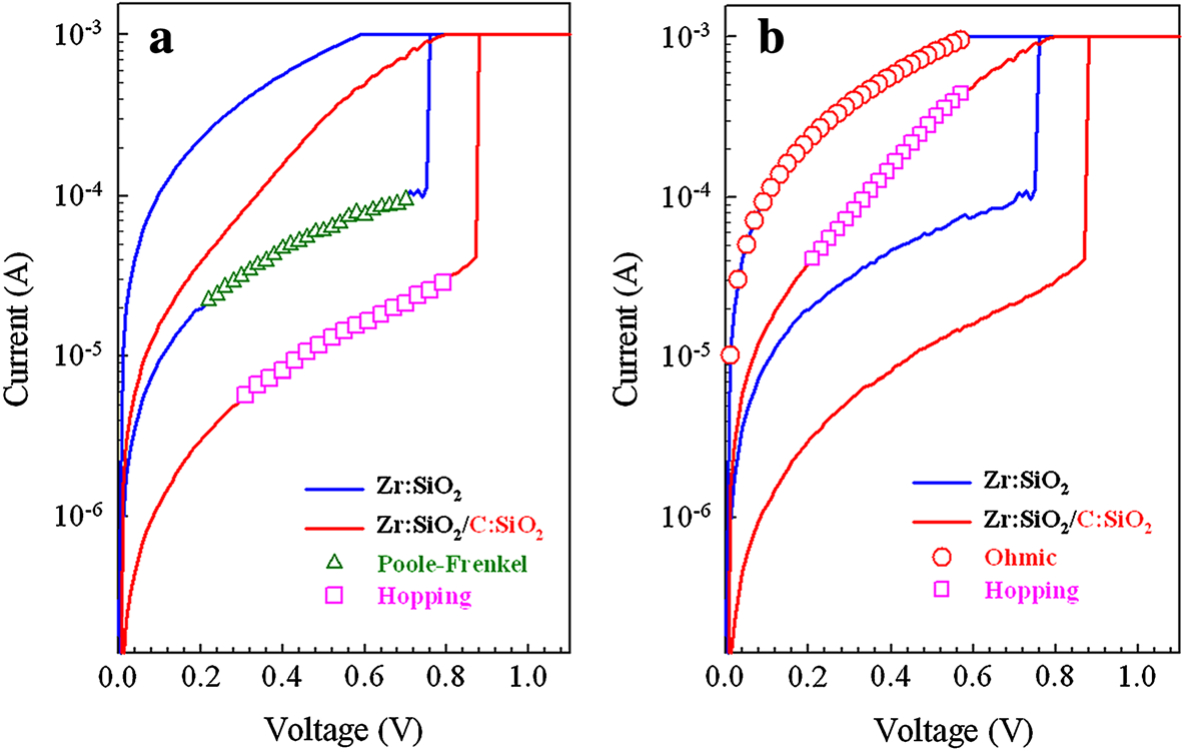
**Current fitting of HRS and LRS of Zr:SiO**_**2 **_**and Zr:SiO**_**2**_**/C:SiO**_**2 **_**RRAM devices, respectively (a, b).** The activation energy of HRS and LRS for hopping conduction is 74.7 and 47.4 meV, respectively.

Through current fitting, we find that both LRS and HRS of double resistive switching layer devices have hopping conduction mechanism, owing to the introduction of carbon element [43], while single resistive switching layer devices exhibit Poole-Frenkel conduction in HRS and Ohmic conduction in LRS (Figure 2).

**Figure 3:**
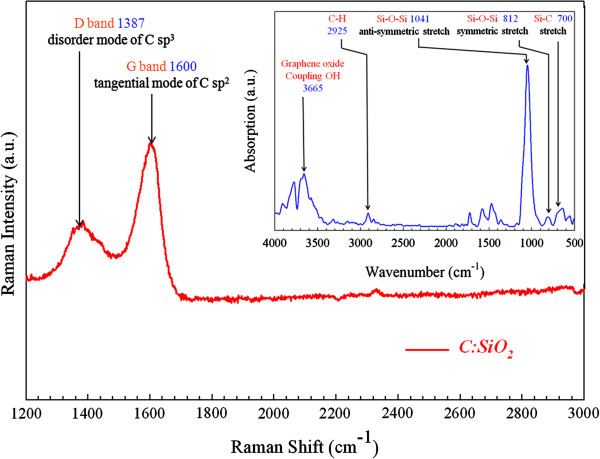
**Raman spectra of C SP**^**2 **^**and C SP**^**3 **^**in C:SiO**_***x***_**film.** It confirms the existence of graphene oxide. The upper inset is the corresponding FTIR spectra, from which graphene oxide coupling OH peak can be observed at the wavenumber of 3,665 cm^-1^.

After that, we utilize material spectra analyses to find out the reason for better performance. First, XPS is applied, from which we obtain the mole fraction of each element in C:SiO_*x*_ and Zr:SiO_*x*_ films. The corresponding element ratios in C:SiO_*x*_ and Zr:SiO_*x*_ are C/Si/O = 7.9:27.32:66.19 and Zr/Si/O = 7.49:26.32:66.19, respectively. To better understand the impact of the inserted C:SiO_*x*_ layer, it is further analyzed by Raman spectroscopy, from which we find typical graphene oxide Raman spectra which is comprised of a higher G band peak and a lower D band peak (Figure 3) [41,47]. In order to further testify the existence of graphene oxide and find its chemical bonding type, FTIR spectroscopy is used to analyze C:SiO_*x*_ film. Graphene oxide coupling OH peak can be observed at the wavenumber of 3,665 cm^-1^, as shown in the top right FTIR spectra of Figure 3.

The resistive switching mechanism in Zr:SiO_*x*_ can be explained by the stochastic formation and rupture of conduction filaments. This is also the reason why we can find Ohmic conduction mechanism in LRS and Pool-Frenkel conduction mechanism in HRS. As in LRS, electrons conduct through metal filaments from the top electrode to the bottom electrode, and in HRS, electrons conduct through shallow defects between the tip of ruptured filament and the bottom TiN electrode. Due to the stochastic formation of conduction filament process, single active layer RRAM device exhibits less stable set voltage and lower degree of uniformity in the reset process.

Comparatively, the C:SiO_*x*_ film works as the switching layer, in which the carrier will hop through the carbon atoms within the carbocycle. If the bottom TiN electrode is applied with a negative bias, oxygen atoms are repelled to the reverse direction of TiN electrode and adsorbed by graphene oxide. With the adsorption of oxygen atoms, carbon-carbon bonds are stretched and carbocycle is enlarged, which results in longer hopping distance of carriers. The adsorption and desorption of oxygen-containing groups are responsible for the resistive switching in graphene oxide-doped silicon RRAM [41-44]. Compared with random formation of conduction filament process, adsorption and desorption of oxygen-containing groups are more stable, as the movement of oxygen-containing groups is much more directional (to graphene oxide). Meanwhile, conduction path always exists, and the difference is hopping distance variation and oxidation rate of graphene oxide. At the top Zr:SiO_2_ layer, the metal filament serves as the conduction way and has the ability of concentrating the electrical field, which facilitates the adsorption and desorption processes of oxygen chemical groups.

**Figure 4:**
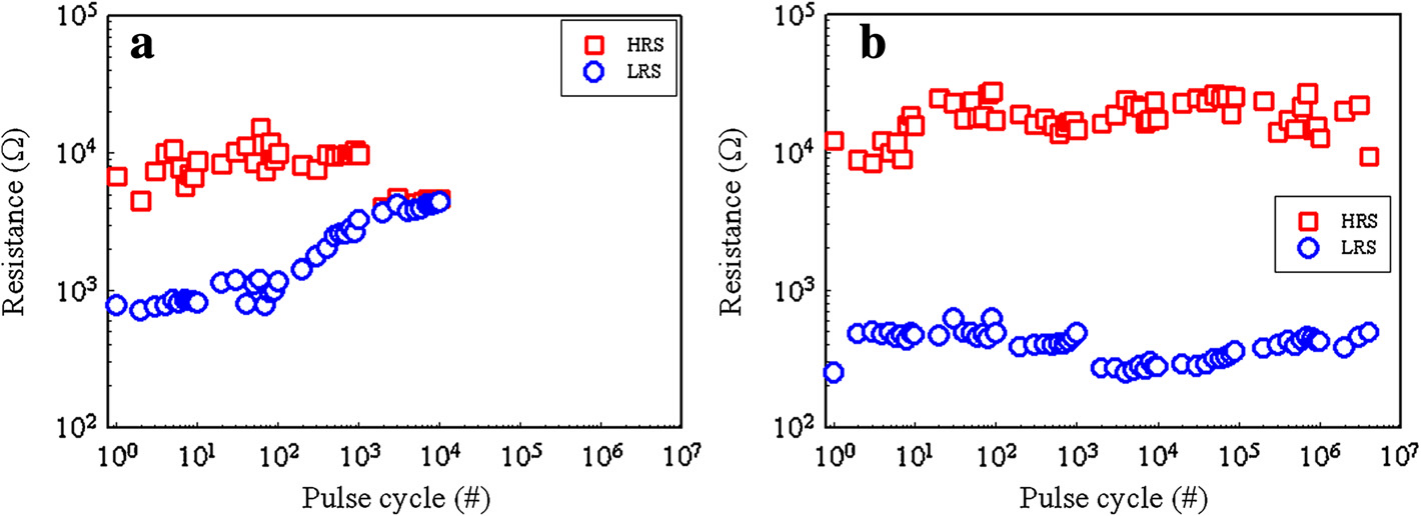
**Endurance characteristics of (a) Pt/Zr:SiO**_**2**_**/TiN structure and (b) Pt/Zr:SiO**_**2**_**/C:SiO**_**2**_**/TiN structure.**

To further evaluate the memory performance, measurement of endurance and retention of both kinds of devices is performed. The retention properties of both types of devices remain stable even after 10^4^ s at 85°C, which satisfy the NVM requirements. The endurance performance is shown in Figure 4. During 10^4^ pulse cycles, the HRS and LRS of Zr:SiO_*x*_ RRAM are short (Figure 4a). While in Zr:SiO_*x*_/C:SiO_*x*_ RRAM device, it exhibits stable HRS and LRS even after more than 10^6^ pulse cycles (Figure 4b).

## Conclusion

In conclusion, by co-sputtering C and Zr with SiO_2_, respectively, we fabricated a double resistive switching layer RRAM, which has significantly outstanding performance. Both FTIR and Raman spectra confirm the existence of graphene oxide in the switching layer of double active layer RRAM devices. Compared with the stochastic formation of conducting filaments, the adsorption and desorption of oxygen atoms from carbocycle work much more stable. This is also the reason why Zr:SiO_*x*_/C:SiO_*x*_ structure has superior switching performance and higher stability.

## Competing interests

The authors declare that they have no competing interests.

## Authors' contributions

RZ and K-CC designed and set up the experimental procedure. T-CC and J-HC planned the experiments and agreed with the paper's publication. T-MT, K-HC, J-CL, and T-FY revised the manuscript critically and made some changes. C-CS fabricated the devices with the assistance of Y-LY and Y-CP. T-JC and S-YH conducted the electrical measurement of the devices. C-HP performed the XPS spectra measurement. Y-TS conducted the FTIR spectra measurement. Y-ES performed the Raman spectra measurement. SMS assisted in the data analysis. All authors read and approved the final manuscript.
